# Role of the Balance of Akt and MAPK Pathways in the Exercise-Regulated Phenotype Switching in Spontaneously Hypertensive Rats

**DOI:** 10.3390/ijms20225690

**Published:** 2019-11-13

**Authors:** Lin Zhang, Yanyan Zhang, Ying Wu, Jingjing Yu, Yimin Zhang, Fanxing Zeng, Lijun Shi

**Affiliations:** 1 Department of Exercise Physiology, Beijing Sport University, Beijing 100084, China; zhanglinbsu@126.com (L.Z.); zhangyanyanbsu1@163.com (Y.Z.); wuyingbsu@163.com (Y.W.);; 2 Key Laboratory of Physical Fitness and Exercise, Ministry of Education, Beijing Sport University, Beijing 100084, China; zhangyiminbsu@163.com; 3 China Institute of Sport and Health Science, Beijing Sport University, Beijing 100084, China; yujingjingbsu@163.com

**Keywords:** hypertension, aerobic exercise, phenotype switching, Akt, MAPK

## Abstract

The mechanisms regulating vascular smooth muscle cell (VSMC) phenotype switching and the critical signal modulation affecting the VSMCs remain controversial. Physical exercise acts as an effective drug in preventing elevated blood pressure and improving vascular function. This study was designed to explore the influence of aerobic exercise on the suppression of VSMC phenotype switching by balancing of the Akt, also known as PKB (protein kinase B) and mitogen-activated protein kinase (MAPK) signaling pathways. Spontaneously hypertensive rats (SHRs) and normotensive rats were subjected to exercise treatment before measuring the vascular morphological and structural performances. Exercise induced reverse expression of VSMC protein markers (α-SM-actin, calponin, and osteopontin (OPN)) in spontaneously hypertensive rats. It is noteworthy that the low expression of phosphorylated Akt significantly decreased the expression of VSMC contractile phenotype markers (α-SM-actin and calponin) and increased the expression of the VSMC synthetic phenotype marker (OPN). However, the MAPK signal pathway exerts an opposite effect. VSMCs and whole vessels were treated by inhibitors, namely the p-Akt inhibitor, p-ERK inhibitor, and p-p38 MAPK inhibitors. VSMC phenotype markers were reversed. It is important to note that a significant reverse regulatory relationship was observed between the expression levels of MAPK and the contractile markers in both normotensive and spontaneously hypertensive rats. We demonstrate that aerobic exercise regulates the VSMC phenotype switching by balancing the Akt and MAPK signaling pathways in SHRs.

## 1. Introduction

According to the mounting evidence, the vascular smooth muscle cell (VSMC) phenotype switching from a contractile (differentiated) phenotype state to a synthetic (proliferated, or dedifferentiated) phenotype state plays a crucial role in a variety of cardiovascular diseases, such as atherosclerosis [1], hypertension [2], coronary heart disease [3], and diabetes [4]. It has been demonstrated that multiple environmental factors, such as growth factors [5], reactive oxygen species (ROS) [6,7], and even mechanical injury [8], are involved in the processes of VSMC growth and phenotype switching. Phenotype switching is characterized by changes in morphology, proliferation, and migration rates, and the expression of different marker proteins [9]. During embryonic development, a synthetic or proliferative phenotype, which has a stronger proliferation and migration ability, is significantly expressed in blood arteries. However, a differentiated or contractile phenotype is predominant in the blood vessels of healthy adults. Importantly, VSMCs undergo a switch from a contractile phenotype to a synthetic or proliferative phenotype after vessel injury. Specifically, under stressed or pathological conditions, highly dedifferentiated contractile cells re-enter the cell cycle, become dedifferentiated, and assume a synthetic or proliferative phenotype. Due to the plasticity, once the injury is resolved, VSMCs return to a non-proliferative, contractile phenotype [10,11,12].

Although important for normal homeostasis, the plasticity of the VSMC phenotype makes these cells particularly susceptible to both physiological and pathological stimuli. Particularly, an abnormal VSMC phenotype has been suggested to play a vital role in the progression of hypertension [13]. It has been demonstrated that one characteristic feature of phenotypically modulated VSMCs is that they express markedly different proteins to perform the VSMC function [14]. Contractile cells express a unique repertoire of contractile markers, such as α-SM-actin [15,16], calponin [17], smooth muscle myosin heavy chain (SMMHC) [18], and SM22α [19]. Osteopontin (OPN) [20] and epiregulin [21] are associated with cell growth, synthesis, proliferation, and migration. Molecular mechanisms underlying the cellular phenotype switching in hypertension are complex and multifactorial. Some vasoactive stimuli, growth factors, epidermal growth factor, and physical factors are involved in the modulation of phenotype switching in VSMCs [22,23]. These factors can activate the membrane receptors and intracellular and extracellular signaling pathways that are important in vascular hypertrophy [24]. For example, some studies have reported that platelet-derived growth factor-BB (PDGF-BB) binds to the PDGF receptor and subsequently activates the intracellular signaling cascades such as protein kinase B (Akt), extracellular signal-regulated kinase (ERK), and p38 mitogen-activated protein kinase (MAPK) pathways [25,26,27]. Akt, also called protein kinase B, is a survival kinase and a main downstream target of the phosphoinositide 3-kinase (PI3K). Mitogen-activated protein kinase (MAPK) cascades are universal triple signaling pathways that include a MAPK kinase kinase (MKKK), a MAPK kinase (MKK), and a terminal MAPK. MAPK comprises the following three major members: ERK, p38 MAPK, and JNK. According to experimental data, extracellular signal-regulated (ERK) and p38 MAPK are involved in VSMC phenotype switching, but Jun N-terminal kinase (JNK) is a different story [28,29]. In our previous study, cross-talk occurred between the Akt and MAPK pathways during phenotype switching in aging and hypertensive rats [30]. 

To our knowledge, the major problem with hypertension is long-term uncontrol of blood pressure. Many pieces of evidence suggest that exercise is considered to be an important treatment for hypertension, and it has a potential benefit on the vascular health in mammals [31,32,33,34]. Exercise training improves the endothelial function and vascular stiffness in small resistance arteries. Decreased oxidative stress and increased NO bioavailability may be important mediators of the effects of exercise [35]. Moreover, our previous study demonstrated that chronic exercise restores the vascular function in mesenteric arteries via remodeling of the Cav1.2 and Kca1.1 channels during hypertension [36]. Exercise is widely used as a kind of “drug,” but it has not shown any side effects such as those resulting from medicine. Significantly, Wen et al. [37] have recently stated that 15 min a day or 90 min a week of moderate-intensity physical exercise is beneficial in terms of life expectancy, even for patients with cardiovascular disease. Vina et al. [38] especially reviewed the molecular mechanisms involved in these beneficial adaptations, including activation of MAPK signaling, AMP-activated protein kinase (AMPK), and reactive oxygen species (ROS).

To date, studies on the regulation of exercise-induced regulation of phenotype switching in VSMCs have not been reported. We are eager to understand the mechanisms behind exercise-induced regulation of phenotype switching. In the present study, we found that exercise is implicated in the expression levels of marker proteins and signaling proteins of the contractile and synthetic phenotypes in spontaneously hypertensive and normotensive rats. To further prove this point, specific inhibitors of Akt, p42/44ERK, and p38MAPK were used to explore the regulation of the Akt and MAPK signaling pathways in phenotype switching in vitro.

## 2. Results

### 2.1. Effects of Aerobic Exercise on the Blood Pressure and Heart Rate of Rats

The experimental data of blood pressure and heart rate before and after exercise treatment are presented in Table 1. All tests were executed at least 24 h after exercise. We tested blood pressure (BP) and heart rate (HR) indirectly through tail arteries in awake rats. Significant differences were observed between sedentary spontaneously hypertensive rats (SHR-SED) and sedentary normotensive rats (Wistar-Kyoto rat-SED, WKY-SED), and spontaneously hypertensive rats showed significantly higher SBP (systolic blood pressure), DBP (diastolic blood pressure), MAP (mean arterial pressure), and HR (heart rate) (*p* < 0.01). Notably, exercise reduced SBP in both SHR-EX (*p* < 0.01) and WKY-EX (*p* < 0.05) groups compared with their matched sedentary groups. In addition, DBP (*p* < 0.05), MAP (*p* < 0.05), and HR (*p* < 0.05) were dramatically declined in the SHR-EX group compared with the SHR-SED group.

### 2.2. Aerobic Exercise Reduces the Wall Thickness of Thoracic Aortas in Spontaneously Hypertensive Rats

**Figure 1:**
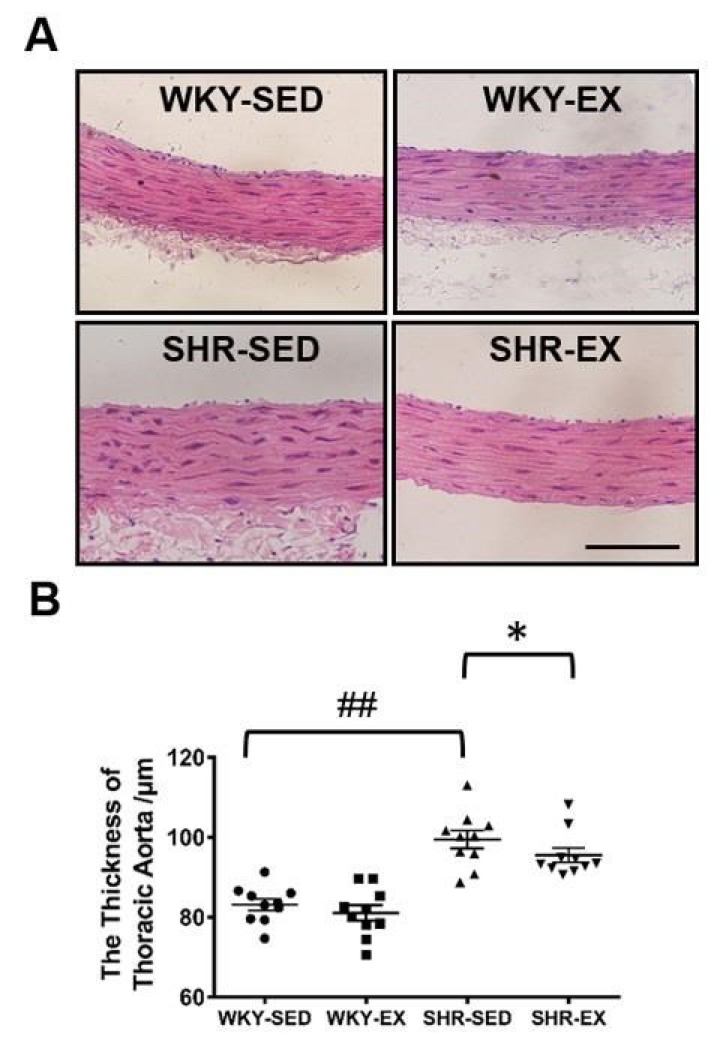
Aerobic exercise modulates VSMC (vascular smooth muscle cell) morphology. Morphological data were detected by hematoxylin-eosin staining. (**A**) The cross-sectional view of the thoracic aorta. The upper left figure depicts WKY-SED (*n* = 10). The lower left box presents SHR-SED (*n* = 10). The upper right box depicts WKY-EX (Wistar-Kyoto rat exercise group) (*n* = 10). The lower right box of A shows SHR-EX (*n* = 10). The analysis results are shown in (**B**). ^##^ *p* < 0.01 (versus WKY-SED), * *p* < 0.05 (versus SHR-SED). Bar = 100 μm.

To explore the potential influence of aerobic exercise on VSMC morphology, we examined the thickness of thoracic aortas (Figure 1). Morphological data showed that the thickness of thoracic aortas was significantly increased in the SHR-SED group versus the WKY-SED group (*p* < 0.01). As expected, we found that physical exercise significantly suppressed the thickening of the blood vessel wall in the SHR-EX group. No significant changes were observed in the WKY rats after exercise treatment.

### 2.3. Aerobic Exercise Changes the VSMC Marker Protein Expression

**Figure 2:**
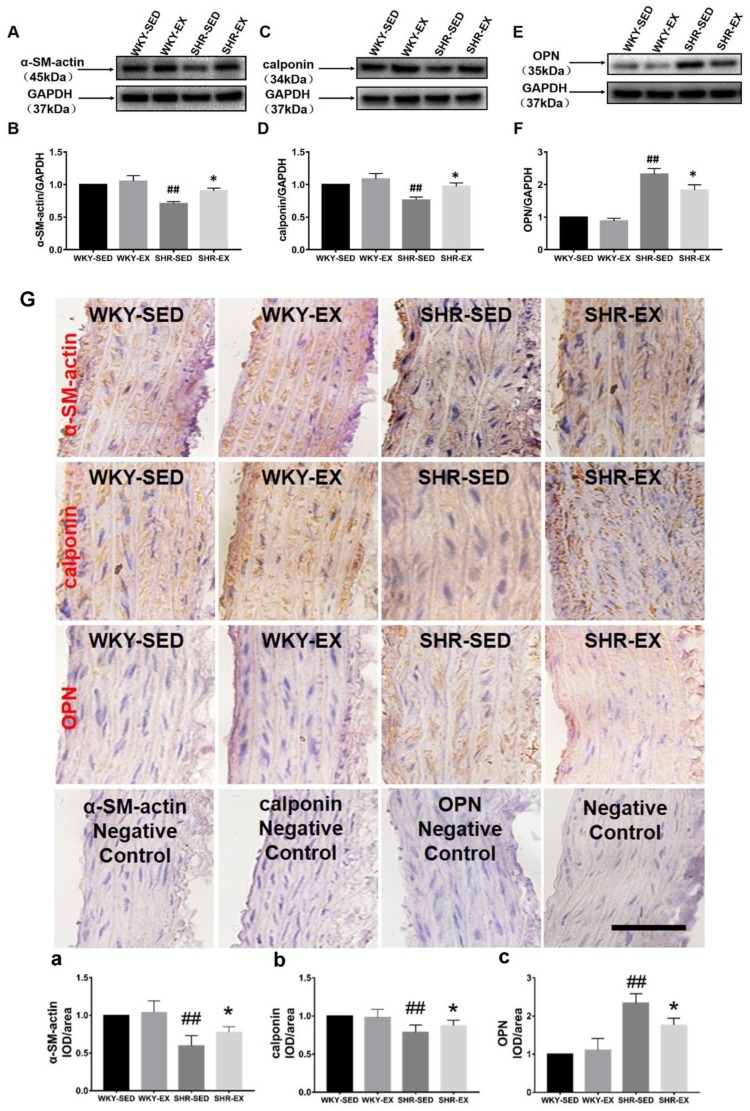
Western blot and immunohistochemistry of VSMC markers with exercise treatment and control. Marker expression levels are shown in (**A**,**C**,**E**) by using Western blot ((**A**): α-SM-actin (alpha smooth muscle actin), (**C**): Calponin; (**E**): OPN (Osteopontin)). Analysis results are shown in (**B**,**D**,**F**) ((**B**): α-SM-actin, (**D**): Calponin; (**F**): OPN). All proteins were normalized to GAPDH which serves as the referential protein. The expression levels of α-SM-actin, calponin, and OPN proteins with and without exercise treatment were measured by immunohistochemistry in (**G**). The analysis results are shown in (**a**–**c**) ((**a**): α-SM-actin, (**b**): Calponin, (**c**): OPN). Negative controls of α-SM-actin, calponin, OPN, and blank are shown in the lower part of the combination image. ^##^ *p* < 0.01 (versus WKY-SED), * *p* < 0.05 (versus SHR-SED). Bar = 50 μm (*n* = 8 in each group).

To explore the functional significance of exercise in VSMC phenotype switching, VSMC protein markers were tested by Western blot and immunohistochemistry assays after exercise treatment (Figure 2). We found that the expression levels of α-SM-actin and calponin, which are contractile markers, were significantly downregulated in spontaneously hypertensive rats. However, the expression level of the synthetic marker OPN was upregulated in spontaneously hypertensive rats. It is interesting to note that exercise training induced an increase in the expressions of contractile markers (α-SM-actin and calponin). Furthermore, physical exercise suppressed the increase in the expression level of the synthetic marker (OPN). These changes in expression levels were revealed both by immunohistochemistry and Western blotting.

### 2.4. Aerobic Exercise Improves the Vasomotor Function of Mesenteric Arteries in Spontaneously Hypertensive Rats

The contractile response of the mesenteric arteries (MAs) third-order branches was evaluated with potassium chloride (KCl, 60 mM) to induce maximal contractions (Kmax). Norepinephrine (NE, 10 μM) was added to the bath to induce vessel constriction. The contractile response of NE was normalized as the maximal response induced by Kmax. The NE-induced contractile response was not shown significantly different between the SHR-SED (189.3% ± 9.7% Kmax) and WKY-SED groups (174.9% ± 8.3% Kmax; *p* > 0.05). However, the tension was dramatically downregulated in the SHR-EX groups (167.7% ± 7.8%) compared with the SHR-SED group (*p* < 0.05). There was no difference in tension between the WKY-EX group (162.1% ± 8.5%) and WKY-SED groups.

**Figure 3:**
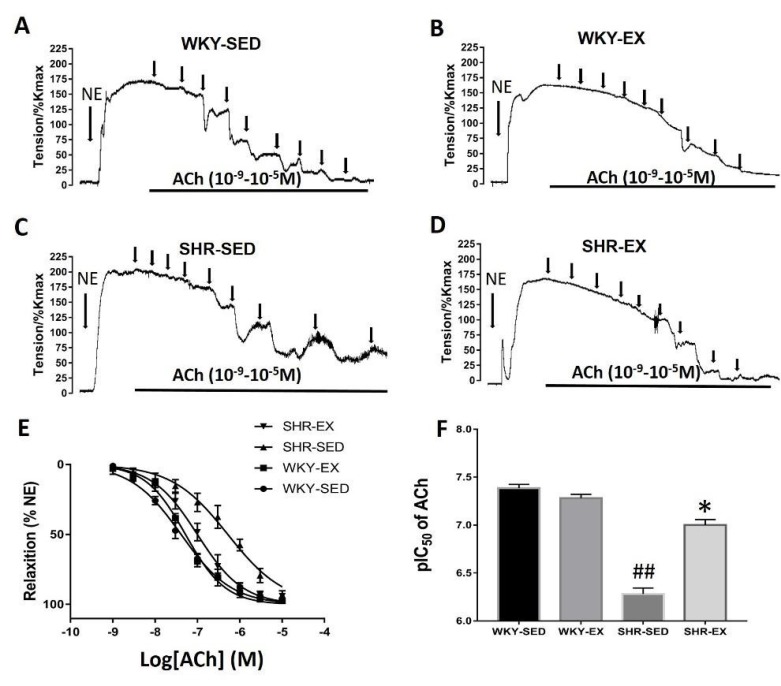
The concentration-response curve to ACh (acetylcholine) in mesenteric arteries. The real-time recording of Ach—induced vasorelaxation in four groups are shown in (**A**–**D**) (ACh: 10^−9^, 3 × 10^−9^, 10^−8^, 3 × 10^−8^, 10^−7^, 3 × 10^−7^, 10^−6^, 3 × 10^−6^, 10^−5^ M). (**E**) shows the coupled curve of Ach—induced concentration-relaxation response curve. The sensitivity of ACh is shown in (**F**). ^##^ *p* < 0.01, compared with WKY-SED; * *p* < 0.05, compared with SHR-SED (*n* = 5 in each group).

To determine the benefit of exercise training on endothelium-dependent vasodilation in MAs, we measured the concentration response curve in MAs induced by acetylcholine (ACh). After the tension equilibrium of the NE (10 μM to induce vasoconstriction, ACh was added in 1/2 log unit steps (Figure 3). The pIC_50_ of the concentration relaxation curve induced by ACh in the SHR-SED group (6.28 ± 0.07) was markedly lower than in the WKY-SED group (7.39 ± 0.04, *p* < 0.05). However, the values of pIC_50_ in the SHR-EX group (7.0 ± 0.06) were higher than in the SHR-SED group (*p* < 0.05). There was no significant difference in pIC_50_ between the WKY-EX and WKY-SED (*p* > 0.05). 

### 2.5. Aerobic Exercise Balances the Functional Role of Akt and MAPK Signaling Pathways in Phenotype Switching

**Figure 4:**
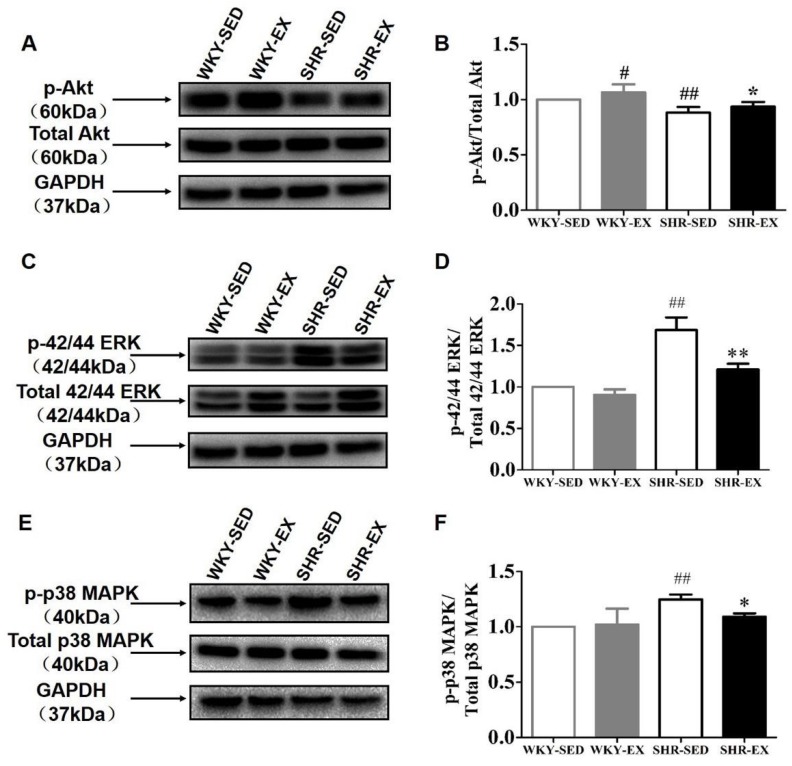
Analyses of signaling pathways in VSMC phenotype regulation. The phosphorylation levels of Akt, known as protein kinase B, and total protein expression are shown in (**A**). The phosphorylation levels of ERK (extracellular signal-regulated kinase) and p38 MAPK (mitogen-activated protein kinase) are presented in (**C**,**E**). (**B**,**D**,**F**) show the analysis data by using GraphPad Prism 7. (**A**,**C**,**E**) were normalized to their total protein. ^##^ *p* < 0.01 (versus WKY-SED), ** *p* < 0.01 and * *p* < 0.05 (versus SHR-SED) (*n* = 8 in each group).

Next, we sought to determine the special function of the Akt and MAPK signaling pathways (Figure 4). The experimental data confirmed our previous hypothesis that the balance of Akt and MAPK signals plays a vital role in VSMC phenotype switching. The levels of the involved proteins, including p-Akt, p-ERK, and p-p38MAPK, were measured in the four groups. In comparison with the WKY-SED group, the SHR-SED group showed significantly decreased expression levels of p-Akt (*p* < 0.01). The expression levels of p-Akt (WKY-EX: *p* < 0.05; SHR-SED: *p* < 0.05) were dramatically upregulated in both the WKY-EX and SHR-EX groups compared with the matched control group (Figure 3A–D). However, an opposite phenomenon was found in the MAPK signaling pathway. We measured the expression levels of p-ERK and p-38MAPK (Figure 3E–H). p-ERK (*p* < 0.01) and p-p38MAPK (*p* < 0.01) were downregulated in sedentary spontaneously hypertensive rats versus sedentary normotensive rats. However, exercise reversed the changes in marker expression caused by high blood pressure. p-ERK (*p* < 0.01) and p-p38MAPK (*p* < 0.05) were upregulated in the SHR-EX group. Taken together, these findings suggest that the balance of Akt and MAPK modulates the VSMC phenotype switching.

### 2.6. Specific Blockers Inhibit the Function of Akt and MAPK Signaling Pathways

**Figure 5:**
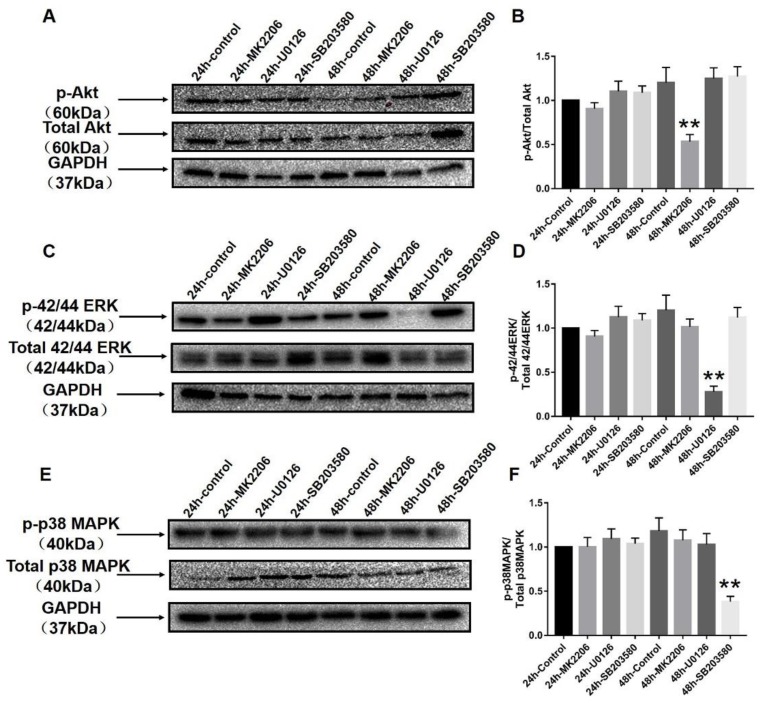
Specific blockers inhibit the function of Akt and MAPK signaling pathways. MK2206, U0126, and SB203580 are specific inhibitors of Akt, ERK, and p38MAPK, respectively, in (**A**,**C**), (**B**,**D**) and (**E**,**F**). (**A**,**C**,**E**) show the efficacy of these blockers at 24 and 48 h time points by using Western blot. After analysis, we can see that p-Akt, p-ERK, and p-p38MAPK were significantly blocked by their blockers at the 48-h time point (**B**,**D**,**F**). Final concentrations are as follows: MK2206 (1 μM), U0126 (10 μM), and SB203580 (10 μM). ** *p* < 0.01 and * *p* < 0.05 versus 24 control group (*n* = 8 in each group).

To verify the underlying molecular mechanisms of Akt, ERK, and p38MAPK, we used their specific inhibitors in vitro cell culture experiments (Figure 5). MK2206, U0126, and SB203580 are the special inhibitors of Akt, ERK, and p38MAPK, respectively. Our previous hypothesis was that the balance of Akt and MAPK signals plays a critical role in VSMC phenotype switching. The smooth muscle cells (SMCs) of the thoracic aorta were treated with specific Akt and MAPK inhibitors. Western blot analysis verified that p-Akt, p-ERK, and p-p38MAPK were successfully knocked down at the 48-h time point in VSMCs by a specific p-Akt inhibitor, a specific p-ERK blocker, and a specific p-p38 MAPK inhibitor, respectively (*p* < 0.01). 

### 2.7. Akt and MAPK Regulate Phenotype Switching of VSMCs in Wistar Rats and In Vitro

**Figure 6:**
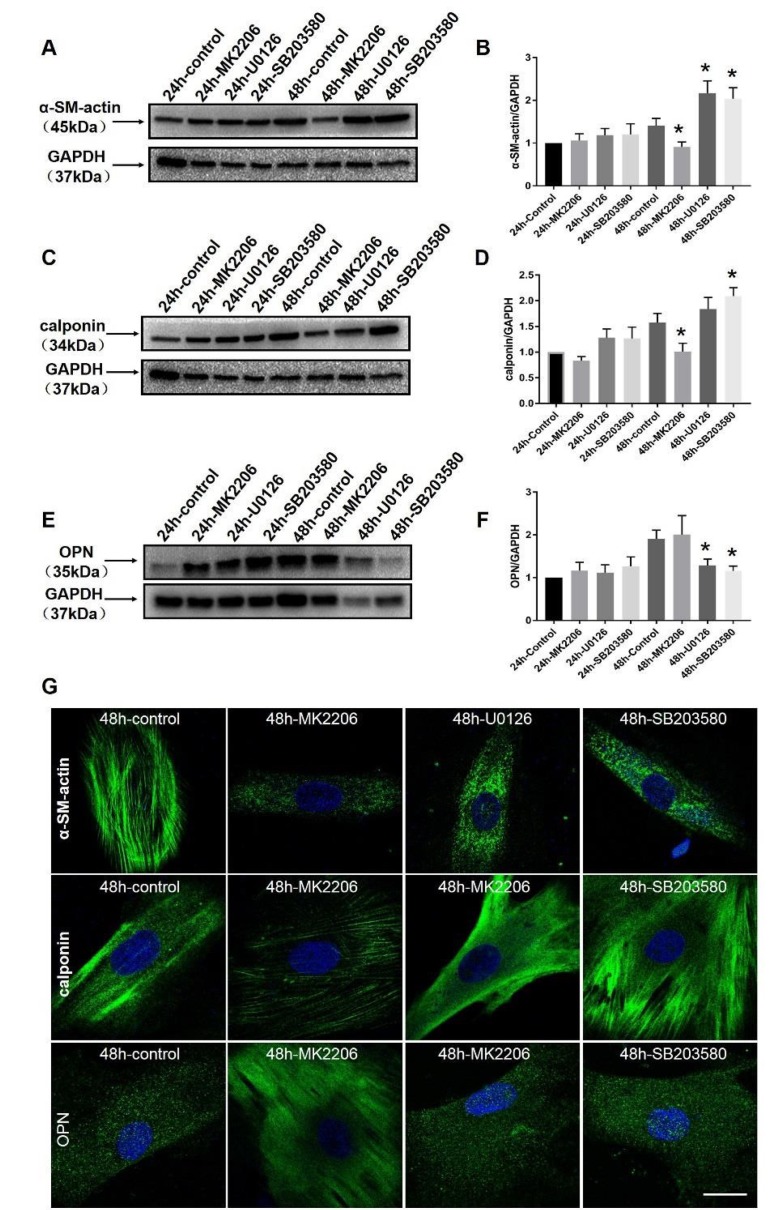
Expression levels of VSMC protein markers after treatments with all inhibitors. Thoracic aortic cells were cultured up to 4–5 generations for official tests. VSMCs were per-incubated by Akt, ERK, and p38 MAPK specific inhibitors. Western blot and immunofluorescence assays were used to test the marker proteins (α-SM-actin, calponin; OPN). Expression levels of α-SM-actin and calponin after treatment with blockers are shown in (**A**–**D**). Images (**E**,**F**) show the expression levels of OPN after treatment with these blockers. Furthermore, immunofluorescence results of marker proteins are shown in (**G**). * *p* < 0.05 versus 24 control group (*n* = 8 in each group). Bar = 25 μm.

To determine the potential role of Akt and MAPK in vascular phenotype switching, we tested the marker proteins of phenotype switching after signal blocker treatments (Figure 6). As mentioned above, MK2206, U0126, and SB203580 were administered into the VSMCs of the rat’s thoracic aorta cultured in vitro. VSMCs of the rat’s thoracic aorta were treated by MK2206, U0126, and SB203580. These blockers were incubated for 24 and 48 h. The protein expression data showed that α-SM-actin and calponin were successfully downregulated in VSMCs at 48 h after MK2206 (*p* < 0.05) treatment, whereas α-SM-actin was dramatically upregulated in VSMCs at 48 h of U0126 (*p* < 0.05) and 48 h of SB203580 (*p* < 0.05) treatments. Calponin only showed an increase after 48 h of SB203580 (*p* < 0.05) treatment. We observed an increase in OPN when the cells were incubated with U0126 (*p* < 0.05) and SB203580 (*p* < 0.05) for 48 h. Immunofluorescence showed the expression levels of α-SM-actin and calponin were significantly increased after 48 h of U0126 and 48 h of SB203580 treatments.

### 2.8. Phenotype Switching of the Pathological State of the Thoracic Aorta and Mesenteric Artery In Vitro

**Figure 7:**
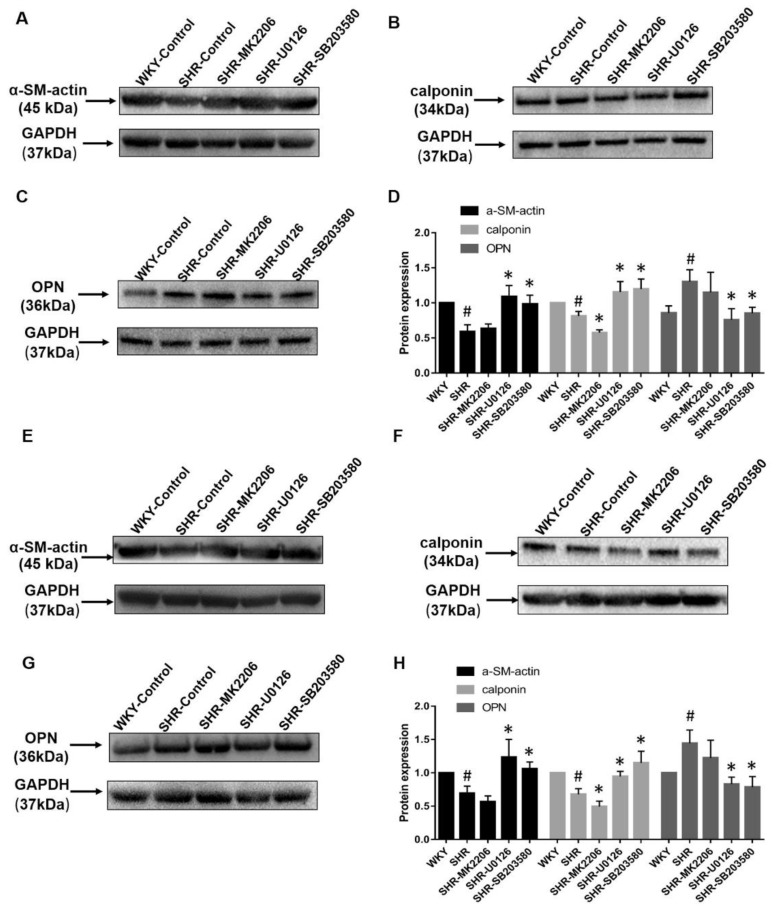
Expression levels of VSMC protein markers after treatments with all inhibitors. Thoracic aortic was per-incubated by Akt, ERK, and p38 MAPK specific inhibitors. Western blot and immunofluorescence assays were used to test the marker proteins (α-SM-actin, calponin; OPN). Expression levels of α-SM-actin and calponin after treatment with blockers are shown in (**A**–**D**). Images (**E**,**F**) show the expression levels of OPN after treatment with these blockers. Furthermore, immunofluorescence results of marker proteins are shown in (**G**). ^#^ *p* < 0.05 versus WKY), * *p* < 0.05 versus SHR. Bar = 25 μm (*n* = 6 in each group).

To further explore whether phenotype switching of VSMCs in SHR is regulated by Akt and MAPK signal pathways, we dissected thoracic aortas and mesenteric arteries from seven-month-old SHRs and age-matched WKY controls (Figure 7). On the one hand, Western blot experiments showed that α-SM-actin and calponin protein expressions were downregulated in SHRs compared with WKYs; however, OPN was upregulated in SHRs compared with WKYs in these two kinds of vessels. On the other hand, the tissue culture technique was used to test the effects of drug interventions on blood vessels. Thoracic aortas and mesenteric arteries from SHRs were treated with MK2206 (p-Akt specific inhibitor), U0126 (p-ERK specific inhibitor), and SB203580 (p-p38 MAPK specific inhibitor). These blockers were incubated for 48 h to achieve inhibition effectiveness according to pre-experiments (Part 2.6). (1) Calponin was markedly downregulated at 48 h after MK2206 (*p* < 0.05) treatment in thoracic aortas and mesenteric arteries from SHRs. (2) α-SM-actin and calponin were dramatically upregulated in these two blood vessels from SHRs by 48 h after U0126 (*p* < 0.05) and 48 h SB203580 (*p* < 0.05) treatments. (3) However, we observed a decrease of OPN when these two kinds of blood vessels were incubated with U0126 (*p* < 0.05) and SB203580 (*p* < 0.05) for 48 h. 

## 3. Discussion

VSMCs are the major components of blood vessels, and they play an important role in the regulation of blood pressure, blood vessel tone, and blood flow distribution. Under pathological conditions, the VSMC phenotype can switch from a contractile (differentiated) state to a synthetic (proliferative) state in vivo and in vitro. Phenotype switching of VSMCs is a hallmark of vascular dysfunction in hypertension [39,40]. Many strategies have been considered to prevent cardiovascular diseases. However, despite a large amount of research on cardiovascular protective mechanisms, exercise is one of the most practical and effective preventive treatments, as Powers et al. [41] have shown. We hypothesized that exercise regulates phenotype switching in VSMCs via the modulation of the Akt and MAKP signaling pathways. In this study, we used in vivo and in vitro models of VSMCs to identify signal regulation in phenotype switching. The present data highlighted the following three important results: 1) Aerobic exercise leads to a dramatic decrease in blood pressure and reverses the vascular morphological changes caused by hypertension. 2) Aerobic exercise regulates VSMC phenotype switching by balancing the Akt and MAPK signal pathways in spontaneously hypertensive rats. 3) According to the in vitro data, the balance of the Akt and MAPK signaling cascades was identified as a critical regulation relationship that is implicated in phenotype switching. 

Currently, there is a wealth of evidence showing that regular physical exercise has beneficial effects in the prevention and treatment of hypertension, including reduction of resting blood pressure, improvement of blood vessel plasticity, and restoration of vascular elasticity [42,43]. Moreover, accordingly, in normotensive subjects, regular exercise reduces the SBP by 3–5 mm Hg and the DBP by 2–3 mm Hg. A recent meta-analysis demonstrated that a mean reduction of 7 mm Hg in SBP and a mean reduction of 5 mm Hg in DBP among hypertensive patients [44]. In the present study, compared with the WKY-SED group, blood pressure values, including SBP, DBP, and MAP, were significantly increased in the SHR-SED group. However, the elevated blood pressure in SHRs can be reduced with exercise treatment. This finding is consistent with previous reports [33,45]. Morphologically, the wall thickness was significantly decreased with exercise treatment in SHRs. Regular aerobic exercise has a beneficial effect in the repair of the vascular condition, and it suppressed the proliferation of VSMCs. 

Many studies have found that hypertension is associated with endothelial dysfunction [46]. Phosphorylation of eNOS causes an increase in the production of nitric oxide (NO) in target tissues, and NO serves as a protective agent against vasoconstriction in a large number of diseases [47,48]. Furthermore, in the cardiovascular circulatory system, endothelium-derived NO plays a main role as an important secondary messenger that regulates physiological and pathological activities [49]. To investigate the endothelial function after exercise treatment, we tested the Ach-induced vasodilation after NE induced pre-contraction. The results showed that compared with the control rats, there was a significant decrease in Ach-induced endothelium-dependent vasodilation in SHRs. However, endothelial induced vasodilation was improved after regulator aerobic exercise.

VSMCs in healthy mammals are a highly specialized cells whose principal function is contraction. However, these cells display remarkable proliferation and migration characteristics and can undergo profound changes in their phenotype during repair of vascular injury, or vascular remodeling in multiple cardiovascular diseases [50]. Most of the VSMCs can switch between a contractile or differentiated state and a synthetic or dedifferentiated phenotype in response to extracellular stimuli [51]. VSMCs are a highly differentiated cell type present in the medial region of arteries and arterioles [52]. It has been reported that hypertension is associated with decreased NO production and activity and increased oxidative stress in blood vessels [53]. Reactive oxygen species (ROS), which are produced by angiotensin II (Ang II), play a critical role in the vascular functions and structures in patients with hypertension [54]. Importantly, the work of Hayashi provides evidence that activation of the Akt pathway is directly involved in maintaining VSMC differentiation under culture conditions [55]. Previous studies have reported the role of the MAPK pathway in physiologically- and pathologically- induced phenotype switching in VSMCs [53]. Results suggest that the activated p38MAPK signaling pathway plays a key role in the process of transformation of the VSMC phenotype from the dedifferentiated to differentiated state [56]. Some findings implied that the proliferative ability of VSMCs was also increased by serum amyloid (SAA) treatment via activation of p38 MAPK [57]. However, existing evidence shows that VSMC phenotypic switching was inhibited by pretreatment with the inhibitor of p38 MAPK [58]. These important studies provide clear evidence suggesting that a fine balance of the Akt and MAPK signaling pathways and regulation are critical for maintaining a healthy phenotype of VSMCs in physiological and pathological conditions.

A number of studies have confirmed that aerobic exercise has benefits in hypertension; however, the underlying mechanisms are complex and still unclear. According to Weinsteina’s research, 10 weeks of aerobic exercise training intervention resulted in increased physical activity and decreased fatigue in individuals with pulmonary arterial hypertension [32]. Interestingly, aerobic exercise has shown beneficial effects in hypertension by improving the redox reaction, especially in the blood vessel wall [35,59]. It is recognized that exercise may potentially prevent hypertension, even hypertensive complications. However, it has not yet been studied whether exercise can modulate the phenotype switching in VSMCs. In this study, we measured two typical contractile proteins, α-SM-actin and calponin, and one synthetic protein OPN in spontaneously hypertensive and normotensive rats. As expected, our results regarding the phenotypic markers in the present experiments are consistent with those in our previous report. These findings suggest that a contractile phenotype was preferentially expressed in healthy VSMCs, whereas a synthetic phenotype was overexpressed in a hypertensive condition. The balance and cross-regulation between Akt and MAPK (ERK and p38 MAPK) signaling cascades regulate phenotype switching in VSMCs. The activities of Akt were dramatically weakened in pathophysiological conditions. The increase and decrease in these two signal proteins were synchronized with those in the contractile proteins (α-SM-actin and calponin). The difference is that ERK and p38 MAPK promote the expression of the synthetic protein (OPN). After eight weeks of aerobic exercise, the phenotype was reversed in spontaneously hypertensive rats, and it showed an increase in the expression levels of contractile proteins and a decrease in the expression levels of synthetic proteins. Furthermore, we observed the significant changes in the Akt and MAPK signaling pathways. In general, exercise not only activates the phosphorylation levels of Akt but also suppresses phosphorylated-ERK and phosphorylated-p38 MAPK to promote the development of a contractile phenotype. 

We further explored the mechanism involved in the balance of the Akt and MAPK signaling pathways in the phenotype switching of VSMCs. VSMCs from thoracic aortic and the whole thoracic aortic and mesenteric arteries from normal and pathological individuals were giving three specific blockers, respectively. MK2206 (Akt inhibitor), U0126 (ERK inhibitor), and SB203580 (p38 MAPK inhibitor) were used to block the Akt, ERK, and p38 MAPK activities. After 48 h of incubation, phosphorylation levels of Akt, ERK, and p38 MAPK were significantly decreased. Phenotypic markers were tested using Western blot. We found that the blockade of Akt significantly inhibited the expression of contractile proteins (α-SM-actin and calponin). Moreover, blocking ERK and p38 MAPK will not only significantly increase the expression levels of contractile proteins, but it will also decrease the OPN expression. Experimental results further indicated that p38 MAPK is pivotal for the VSMC phenotype switching. Overall, a series of evidence demonstrated the important role of the balance of Akt and MAPK signaling pathways in the process of phenotype switching in VSMCs. Our data specifically show that p38 MAPK is involved in simultaneous regulation of expression of the following three proteins: α-SM-actin, calponin, and OPN. 

## 4. Materials and Methods 

### 4.1. Animals and Exercise Protocol

Adult three-month-old male spontaneously hypertensive rats (SHR) and Wistar-Kyoto rats (WKY) were studied in an exercise protocol. We used seven-month-old SHRs and age-matched WKY rats in the organ culture. All the rats were purchased from Vital River Laboratory Animal Technology Temperature (Beijing, China) and maintained in a 12:12 h light-dark cycle at a temperature of 23–25 °C and a humidity level of 40–60% with free access to standard rodent chow and water. Three-month-old SHRs and age-matched WKY rats were randomly assigned to sedentary groups (SHR-SED, WKY-SED) and aerobic exercise training groups (SHR-EX, WKY-EX). Each exercise-trained group ran 60 min per day, five days per week for eight weeks (speed: 20 m/min). 

### 4.2. Blood Pressure and Heart Rate Measurements

SBP, DBP, MAP, and heart rate were measured in warmed, restrained, conscious rats using the artery tail-cuff method (BP–2010A, Softron Biotechnology Ltd., Beijing, China). To adapt the participants to the procedure, the pressure was measured on several occasions during the treatment period. A minimum of 10 measurements was performed per rat, with the mean values within a range of 5 mm Hg representing the recorded blood pressure level. 

### 4.3. Material Preparation

At the end of the eight-week exercise treatment periods, all the animals were anesthetized with sodium pentobarbitone (50 mg·kg^−1^, intraperitoneal). Tissue samples were harvested from the euthanized rats of each group, and all arteries were cleaned of the adhering fat and connective tissue gently. The experimental protocols using laboratory animals were approved of by the ethical committee of Beijing Sport University. The trial was approved by the Scientific Research Internal Review Board of Beijing Sport University (1 Jan. 2018) and the authorization number is 2017003A. Written informed consent was obtained from all participants. The experiments were performed in accordance with Chinese animal protection laws and institutional guidelines.

### 4.4. Histological Assays

For morphological assays, thoracic aortas were carefully dissected from the connective tissue, avoiding mechanical stretch, and they were perfused with 4% paraformaldehyde for 12 h. Subsequently, the vessels were rinsed with phosphate-buffered solution, dehydrated with ethanol, and then embedded in paraffin. Paraffin sections were cut at a thickness of 4 μm. These tissue segments were stained with hematoxylin and eosin (HE), and images were obtained using an inverted microscope (IX71-F22PH, OLYMPUS, Tokyo, Japan) connected to a computer. For the quantitative analysis on the thicknesses were calculated by using Image-Pro Plus 6.0 (MEDIA CYBERNETICS, Rockville, MD, USA).

### 4.5. Immunohistochemistry

Paraffin was removed by xylene from serial sections of 4 μm thickness. The segments were rehydrated in graded ethanol, and then they were pretreated with 0.01 M citric acid buffer (pH = 6) for 20 min in a thermostatic water bath (99 °C). The materials were pre-incubated with 3% hydrogen peroxide in phosphate-buffered saline (PBS) solution for 5 min to inactivate of endogenous peroxidase. The segments were incubated in 0.3% Triton X-100 (15 min), and then the sections were washed in 0.01 M PBS (3 × 5 min). The sections were blocked with 5% normal goat serum in PBS solution for 20 min. The samples were incubated with anti-alpha smooth muscle actin antibody (ab5694, Abcam, Cambridge, MA, USA, 1:200), anti-osteopontin antibody monoclonal primary antibody (ab63856, Abcam, Cambrige, MA, USA, 1:100), and antibody anti-calponin 1 antibody (sc58707, Santa Cruz Biotechnology, Dallas, TX, USA, 1:200). Incubation was carried out overnight at 4 °C in the refrigerator. The sections were then incubated with biotin-conjugated secondary antibody anti-rabbit IgG (SA00001-2, Proteintech Group, Rosemont, IL, USA) and antibody anti-mouse IgG (SA00001-1, Proteintech Group, Rosemont, IL, USA) at a concentration of 1:200 in PBS for 1 h. The sections were washed three times with PBS. The bound complexes were visualized by the application of a 0.05% solution of 3,3′-diaminobenzidine solution and counterstained with Harris hematoxylin. The serial segments were treated with rabbit IgG and mouse IgG at a concentration of 1:200 in place of the primary antibody in control samples. The images were obtained by an inverted microscope (IX71-F22PH, OLYMPUS, Tokyo, Japan) connected to a computer.

### 4.6. Wire Myography of Mesenteric Arteries

All the rats were anesthetized with sodium pentobarbitone (60 mg/kg, intraperitoneal) at the end of the eight-weeks exercise training. The mesenteric arteries were dissected from rats and transferred into cold PSS (131.5 mM NaCl, 5 mM KCl, 1.2 mM NaH_2_PO_4_, 1.2 mM MgCl_2_, 2.5 mM CaCl_2_, 11.2 mM glucose, 13.5 mM NaHCO_3_, and 0.025 mM EDTA, and they were gassed with 95% O_2_ and 5% CO_2_, regulated to pH = 7.4) rapidly. Scissors were used to remove the connective tissues gently and cut the third-order branches of mesenteric arteries into ~2-mm cylindrical segments, trying to eliminate blood clots inside the artery, if present. The arteries were mounted with a multi-myograph system (620 M, DMT, Denmark). After equilibration 30 min, a normalization procedure was carried out using LabChart software (AD Instruments, UK). KCl (60 mM) was applied to induce the maximal contraction. The endothelium-dependent relaxation response to acetylcholine (ACh, 10^−9^–10^–5^ M) was measured as MAs pre-constricted with norepinephrine (NE, 10 μM).

### 4.7. Western Blot Analysis

Thoracic aortas were homogenized on ice with lysis buffer, and the supernatant was collected after centrifugation. The protein concentration was determined by the Bradford method using bovine serum albumin as a standard. Equal amounts of proteins were separated by 10% SDS-polyacrylamide gel electrophoresis gels and electrophoretically transferred to a polyvinylidene fluoride (PVDF) membrane. After blocking with bovine serum albumin, the membrane was washed and incubated overnight at 4 °C with primary antibody against GAPDH (sc32233, Santa Cruz Biotechnology, Dallas, TX, USA, 1:500). On the second day, the proteins were incubated with the secondary antibody, followed by autoradiography using the ChemiDoc XRS+ System (Bio-Rad Laboratories, Hercules, CA, USA). The band intensities were quantified using Image Lab Software (Bio-Rad Laboratories, Hercules, CA, USA), and they are expressed as a percentage of GAPDH in each lane. The protein expression of p-Akt/Akt (anti-phospho-Akt (Ser473) antibody, cell signaling, catalog No. 9271s; anti-Akt antibodies, cell cignaling, catalog No. 9272s), p-ERK/ERK (anti-phospho-ERK antibody, cell signaling, catalog No. 9101s; anti-ERK antibody (cell signaling, catalog No. 9102s), p-p38 MAPK/p38 MAPK (anti-phospho-P38 MAPK antibody, cell signaling, catalog No. 9215s; anti-p38 MAPK antibody, cell signaling, catalog No. 9212s), α-SM-actin, calponin, OPN were tested. 

### 4.8. Cell Culture and Organ Culture 

VSMCs were isolated from thoracic aortas of 12-week-old Wistar rats. The adventitia and the intima were removed from the medial layer, and cells were grown from the medial layer explants in Dulbecco’s modified eagle medium (DMEM, Gibco, Carlsbad, CA, USA) with 10% fetal bovine serum (FBS, Paragon Biotech, Baltimore, MD, USA) and a 1% penicillin/streptomycin solution (Gibco) in an incubator containing 5% CO2 and 95% air at 37 °C. When cells were grown to 70–80% confluence, the explants were removed and the VSMCs were cultured in the same solution. When VSMCs were in the fifth passage, they were treated with an Akt inhibitor (MK2206: 1 μM), an ERK inhibitor (U0126: 10 μM), and a p38 MAPK inhibitor (SB203580: 10 μM) for 24 and 48 h. Signal proteins were harvested from freshly cultured VSMCs to determine the efficacy of specific blockers. VSMC protein markers were tested at each indicated time point using immunofluorescence and Western blot.

Thoracic aortas and mesenteric arteries were acutely dissected from WKY rats (seven months of age, *n* = 6) and SHRs (seven months of age, *n* = 6), the surrounding fatty tissue was removed from arteries gently. Smooth and clean blood vessels were cultured by placing arteries in serum-free DMEM-F12 culture media (Gibco), with added 100 U/mL penicillin, 100 mg/mL streptomycin at 37 °C in a humidified atmosphere with 5% CO_2_. WKY rats without any treatment were the control group. Thoracic aortas and mesenteric arteries from SHRs were treated with an Akt inhibitor (MK2206: 1 μM), an ERK inhibitor (U0126: 10 μM), and a p38 MAPK inhibitor (SB203580: 10 μM) for 48 h. Phenotypic protein markers (contractile proteins: α-SM-actin and calponin; synthetic protein: OPN) were measured using Western blot and immunofluorescence.

### 4.9. Immunofluorescence

Primary cultured smooth muscle cells of thoracic aortas were fixed with 4% paraformaldehyde in PBS for 30 min. Following a wash in PBS, VSMCs were permeabilized with 0.1% Triton X-100, blocked with 5% BSA (Bull Serum Albumin) for 1 h, and incubated overnight with primary antibody (anti-α-SM-actin, anti-calponin, and anti-OPN antibody) at 4 °C. The second day, after a wash in PBS, VSMCs were then incubated with Alex Fluor 488 Goat anti-Mouse IgG (H+L) Secondary Antibody and Alex Fluor 488 Goat anti-Rabbit IgG (H+L) Secondary Antibody (1:1000, Molecular Probes) at room temperature for 1 h. VSMCs were sealed with Anti-Fade Solution (Prolong^TM^ Gold Antifade Mountant with DAPI, Molecular Probes). Images were acquired using laser-scanning confocal microscope.

### 4.10. Statistical Analysis

Results are expressed as mean ± SEM. Normality tests were used to determine the normal distribution of the data; and when the data were not normally distributed, log-transformation was applied. Two-way analysis of variance (ANOVA, hypertension × exercise) was used in this study. For in vitro experiments, an unpaired *t*-test was used to compare two groups of the same observation time point. Values of *p* < 0.05 were considered statistically significant. All analyses were performed using GraphPad Prism 7 and results were obtained using GraphPad Prism 7.

## 5. Conclusions

Taken together, this study explores the underlying mechanisms responsible for the exercise-mediated anti-hypertensive effects. Moreover, in this research, we present compelling evidence that exercise has been shown to exert a beneficial influence to phenotype switching via the regulation of the balance of Akt and MAPK signal pathways in VSMC. Mechanistically, our experimental results illustrate the role of the balance of Akt and MAPK signal pathways together to modulate VSMC phenotype switching.

## Figures and Tables

**Table 1:** Aerobic exercise modulates blood pressure (BP) and heart rate (HR).

Items	Stage	WKY-SED (*n* = 12)	WKY-EX (*n* = 12)	SHR-SED (*n* = 12)	SHR-EX (*n* = 12)
SBP	Initial	134.9 ± 3.6	134.9 ± 3.4	191.6 ± 7.3 ^##^	192.1 ± 3.9
	Final	137.6 ± 3.8	131.2 ± 5.3 ^#^	195.8 ± 9.02 ^##^	182.9 ± 3.6 **^$$^
DBP	Initial	93.7 ± 4.6	94.0 ± 2.5	145.6 ± 4.2 ^##^	145.4 ± 2.4
	Final	98.2 ± 2.8	95.1 ± 3.8	148.4 ± 3.7 ^##^	139.6 ± 2.7 *^$^
MAP	Initial	107.4 ± 2.8	107.6 ± 3.9	160.9 ± 3.2 ^##^	161.0 ± 2.8
	Final	111.3 ± 4.03	107.1 ± 4.3	164.9 ± 5.6 ^##^	154.2 ± 5.0 *^$^
HR	Initial	355.4 ± 9.6	359.4 ± 7.3	412.1 ± 7.0 ^##^	418.5 ± 10.1
	Final	364.1 ± 8.0	358.5 ± 6.4	421.2 ± 9.0 ^##^	400.2 ± 11.4 *^$^

^#^*p* < 0.05 and ^##^ *p* < 0.01, compared with WKY-SED (Wistar-Kyoto rat sedentary group); * *p* < 0.05 and ** *p* < 0.01, compared with SHR-SED (spontaneously hypertensive rat sedentary group); ^$$^ *p* < 0.01 and ^$^ *p* < 0.05, compared with initial. SBP: Systolic blood pressure; DBP: Diastolic blood pressure; MAP: Mean arterial pressure; and HR: Heart rate.

## Abbreviations

ACh	Acetylcholine
AMPK	AMP-activate protein kinase
BP	Blood pressure
DBP	Diastolic blood pressure
eNOS	Endothelial nitric oxide synthase
ERK	Extracellular regulated protein kinase
MAP	Mean arterial pressure
MAPK	Mitogen-activated protein kinase
NE	Norepinephrine
NO	Nitric oxide
PI3K	Phosphatidylinositol 3-kinase
PDGF–BB	Platelet derived growth factor-BB
PKB	Protein kinase B
ROS	Reactive oxygen species
SBP	Systolic pressure
SHR	Spontaneously hypertensive rat
VSMC	Vascular smooth muscle cell
WKY	Wistar-Kyoto rat
